# Peripheral Blood CD8^+^ T-Lymphocyte Immune Response in Benign and Subpopulations of Breast Cancer Patients

**DOI:** 10.3390/ijms25126423

**Published:** 2024-06-11

**Authors:** Marek Lenárt, Peter Bober, Miroslav Marcin, Soňa Tkáčiková, Mária Kacírová, Michal Alexovič, Dávid Tóth, Natália Madárová, Jozef Radoňak, Peter Urdzík, Ján Fedačko, Ján Sabo

**Affiliations:** 1 1st Department of Surgery, Faculty of Medicine, University of Pavol Jozef Šafárik and UNLP in Košice, Trieda SNP 1, 04011 Košice, Slovakia; marek.lenart@student.upjs.sk (M.L.); natalia.madarova@student.upjs.sk (N.M.); jozef.radonak@upjs.sk (J.R.); 2 Department of Medical and Clinical Biophysics, Faculty of Medicine, University of Pavol Jozef Šafárik in Košice, Trieda SNP 1, 04011 Košice, Slovakia; miroslav.marcin@upjs.sk (M.M.); sona.tkacikova@upjs.sk (S.T.); michal.alexovic@upjs.sk (M.A.); 3 Center of Clinical and Preclinical Research MEDIPARK, Faculty of Medicine, University of Pavol Jozef Šafárik in Košice, Trieda SNP 1, 04011 Košice, Slovakia; maria.kacirova@upjs.sk (M.K.); jan.fedacko@upjs.sk (J.F.); 4 Department of Gynaecology and Obstetrics, Faculty of Medicine, University of Pavol Jozef Šafárik and UNLP in Košice, Trieda SNP 1, 04011 Košice, Slovakia; david.toth@upjs.sk (D.T.); peter.urdzik@upjs.sk (P.U.)

**Keywords:** breast cancer, CD8^+^ T lymphocytes, RNA degradation, granzymes

## Abstract

Peripheral blood CD8^+^ T lymphocytes play a crucial role in cell-mediated immunity and tumor-related immune responses in breast cancer. In this study, label-free quantification analysis and gene set enrichment analysis (GSEA) of CD8^+^ T lymphocytes in the peripheral blood of benign patients and patients with different breast cancer (BC) subtypes, i.e., luminal A, luminal B, and triple-negative breast cancer (TNBC), were performed using nano-UHPLC and Orbitrap mass spectrometry. Differential protein expression in CD8^+^ T lymphocytes revealed significant downregulation (log_2_ FC ≥ 0.38 or ≤−0.38, adj. *p* < 0.05), particularly in proteins involved in cytotoxicity, cytolysis, and proteolysis, such as granzymes (GZMs) and perforin 1 (PRF1). This downregulation was observed in the benign group (GZMH, GZMM, and PRF1) and luminal B (GZMA, GZMH) subtypes, whereas granzyme K (GZMK) was upregulated in TNBC in comparison to healthy controls. The RNA degradation pathway was significantly downregulated (*p* < 0.05, normalized enrichment score (NES) from −1.47 to −1.80) across all BC subtypes, suggesting a potential mechanism for regulating gene expression during T cell activation. Also, the Sm-like proteins (LSM2, LSM3, and LSM5) were significantly downregulated in the RNA degradation pathway. Proteomic analysis of CD8^+^ T lymphocytes in peripheral blood across different breast cancer subtypes provides a comprehensive view of the molecular mechanisms of the systemic immune response that can significantly contribute to advancements in the diagnosis, treatment, and prognosis of this disease.

## 1. Introduction

The most prevalent type of cancer, especially affecting women, is breast cancer (BC). Approximately 2.3 million instances of breast cancer were detected globally in 2020, with 685,000 deaths attributed to the disease. By 2020, 7.8 million women had survived the disease in the previous five years, making it the most common cancer worldwide [1]. Human epidermal growth factor 2 (HER2) and estrogen (ER) or progesterone receptors (PR) molecular markers are used to classify BC into several subtypes: 70% of patients are hormone receptor (HR) positive/HER2 negative, 15–20% are HER2 positive, and 15% are triple-negative breast cancer (TNBC) [2]. Many patients have poor prognoses as a result of not responding to systemic therapies such as hormone therapy, chemotherapy, or molecular targeted therapy during their treatments [3,4]. In light of this, researchers have looked at developing novel, efficient therapies, such as immunotherapy and metabolic therapy [5,6]. The results of the studies indicate that interactions and crosstalks between immune and tumor cells have a significant impact on the development of the tumor and its subsequent progression towards metastasis [7].

An integral part of the immune defense system is peripheral blood mononuclear cells (PBMCs). T lymphocytes are among these cells that present mobile cells, continuously recirculating between the blood and tissues, returning to the blood via the lymphatic system [8]. One of the most important parts of the adaptive immune system that responds to malignancies is the CD8^+^ T lymphocyte. Naive CD8^+^ T cells get activated and differentiate into effector cells in response to antigen stimulation. Following antigen clearance, a small fraction of memory cells may form [9,10].

Studies on cytotoxic T cells (CTLs) associated with breast cancer have demonstrated their memory phenotype, proapoptotic and lytic activities [11] in the majority of this cell population, especially in high-grade lesions [12]. The cytotoxic protease family known as granzymes (Gzms), which comprises GZMA, GZMB, GZMH, GZMK, and GZMM, is crucial for the elimination of target cells by CTLs [13]. CTLs employ granules (secretory vesicles) to store cytotoxic molecules. After the T cell receptor (TCR) on the target cell is ligated, these molecules are released toward the immunological synapse [14,15]. Together with Gzms, cytotoxic granules also include granulysin and perforin. Granulysin may rupture intracellular microbial membranes [16] as well the pore-forming protein perforin that damages cell membranes and makes it easier for Gzms to enter the cytoplasm of target cells [17]. Every one of the granzyme proteases acts on specific and unique substrates to initiate cell death in the target cell [18,19].

The regulating of T cell-mediated immunity plays a central role in the RNA metabolism that closely correlates to RNA decay as well as the regulation of gene expression during phase transition, i.e., development, resting, activation and effector function T cells. RNA decay is a complex process that involves various enzymes and RNA-binding proteins. RNA may degrade by different pathways, such as endonucleolytic decay which entails RNA cleavage at specific internal locations, whereas exonucleolytic decay includes the gradual nucleotide loss from the RNA molecule ends [20].

Peripheral blood collection is a minimally invasive method that may be repeated throughout therapy without discomfort, unlike biopsy. Several studies have reported changes in the expression of CD8^+^ tumor-infiltrating lymphocytes (TILs) of patients with different subtypes of breast cancer [21,22]. However, we have found no study based on comparative proteomics of CD8^+^ T lymphocytes in peripheral blood in different subtypes of breast cancer.

## 2. Results

### 2.1. Proteomic Analysis Using Orbitrap Exploris™ 480 MS Coupled to an UHPLC

A total of 47 blood samples, including eight from benign patients, eight from patients with luminal A, eight from patients with luminal B, three from patients with TNBC, and twenty from healthy controls, were used. Utilizing a quantitative label-free method (ion intensity), proteomic analysis was performed between healthy controls and benign and breast cancer subpopulations of the CD8^+^ T lymphocytes isolated from peripheral blood using nano-UHPLC and an Orbitrap mass spectrometer.

### 2.2. Label-Free Quantitative Proteomic Analysis of CD8^+^ T Lymphocytes in the Peripheral Blood Samples

**Figure 1:**
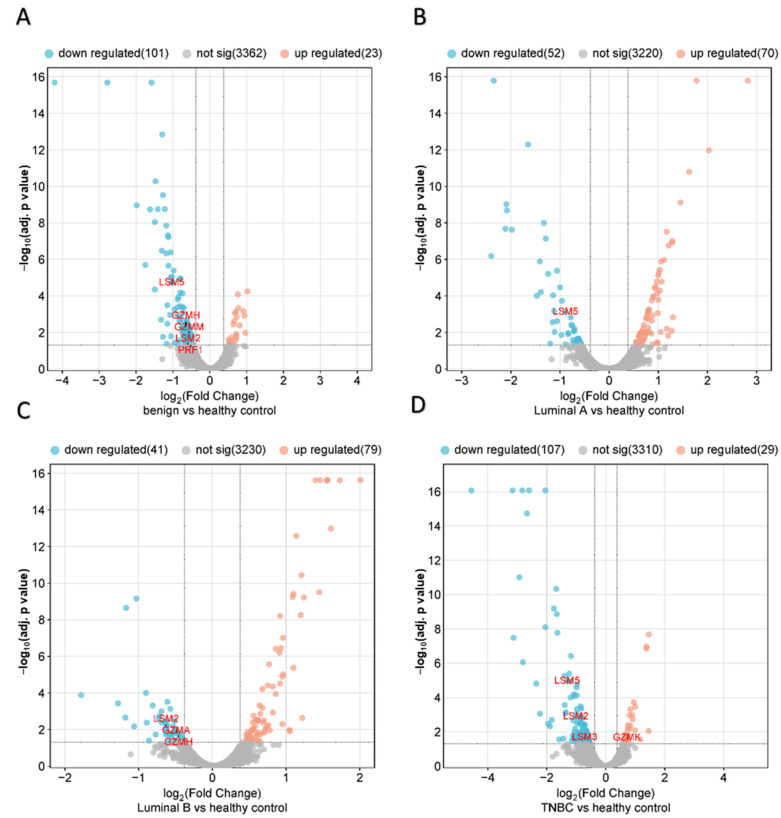
Volcano plots of the differentially expressed proteins in CD8^+^ T lymphocytes between the (**A**) benign, (**B**) luminal A, (**C**) luminal B, and (**D**) TNBC vs. healthy control. An online web tool (SRPlot) was used to create volcano charts (https://www.bioinformatics.com, accessed on 13 May 2024) (adjusted *p*-value less than 0.05 and log_2_ FC ≥ 0.38 or ≤−0.38).

The differential expression of proteins in the CD8^+^ T lymphocytes of benign and BC subtypes (luminal A, luminal B, & TNBC) in comparison to healthy subjects was analyzed based on quantitative protein profiling using volcano plots. Among the 3486 total proteins identified/quantified in benign samples versus the healthy control, 124 proteins were significantly altered (log_2_ FC ≥ 0.38 or ≤−0.38, adjusted *p*-value < 0.05), of which there were 23 proteins that were upregulated and 101 proteins that were downregulated in comparison to the healthy control (Figure 1A, Table S1). Similarly, among the 3342 total proteins identified/quantified in the luminal A group, 122 proteins were significantly altered (log_2_ FC ≥ 0.38 or ≤−0.38, adjusted *p*-value < 0.05), of which there were 70 proteins that were upregulated and 52 proteins that were downregulated in comparison to the healthy control (Figure 1B, Table S1). Next, among the 3350 total proteins identified/quantified in the luminal B group, 120 proteins were significantly altered (log_2_ FC ≥ 0.38 or ≤−0.38, adjusted *p*-value < 0.05), of which there were 79 proteins that were upregulated and 41 proteins that were downregulated in comparison to the healthy control (Figure 1C, Table S1). Lastly, out of the 3446 total proteins identified/quantified in the TNBC group, 136 proteins were significantly altered (log_2_ FC ≥ 0.38 or ≤−0.38, adjusted *p*-value < 0.05), with 29 of them being upregulated and 107 downregulated in comparison to the healthy control (Figure 1D, Table S1).

**Figure 2:**
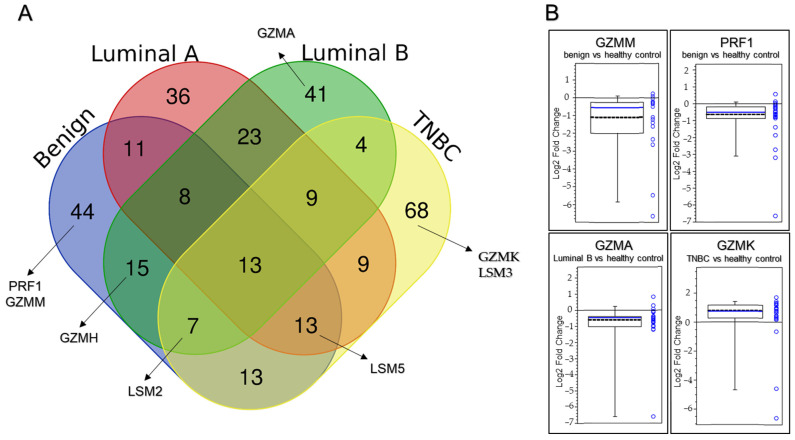
(**A**) Venn diagram showing overlapping and unique subtype-specific proteins. Blue: benign; red: luminal A; green: luminal B; Yellow: TNBC. PRF1, GZMA, GZMH, GZMK and GZMM proteins that were significantly altered (log_2_ FC ≥ 0.38 or ≤−0.38, adjusted *p*-value < 0.05) and participated in T cell-mediated cytotoxicity and cytolysis. LSM2, LSM3, and LSM5 proteins were significantly altered (log_2_ FC ≥ 0.38 or ≤−0.38, *p*-value < 0.05) and participated in the RNA catabolic (degradation) process. (**B**) Box plot of differentially expressed proteins PRF1, GZMH and GZMM that were downregulated in the benign group and GZMK that was upregulated in TNBC.

We used a Venn diagram to show overlapping and unique subtype-specific proteins (PRF1, GZMA, GZMH, GZMK and GZMM) that were significantly altered (log_2_ FC ≥ 0.38 or ≤−0.38, adjusted *p*-value < 0.05) and participated in T cell-mediated cytotoxicity, cytolysis and proteolysis. Among them, PRF1, GZMH and GZMM in the benign group were downregulated in the following order: log_2_ FC = −0.52, −0.65 and −0.56 (Figure 1A and Figure 2A). Simillarly, GZMA and GZMH in luminal B (Figure 1C and Figure 2A) were downregulated in the following order: log_2_ FC = −0.49 and −0.46. In contrast, GZMK (log_2_ FC = 0.72) in the TNBC group (Figure 1D and Figure 2B) was upregulated. We also focused on proteins (LSM2, LSM3 and LSM5) that were significantly altered (log_2_ FC ≥ 0.38 or ≤−0.38, adjusted *p*-value < 0.05), participating in the RNA catabolic (degradation) process. LSM2 was downregulated in the following groups: log_2_ FC = −0.59 (benign), −0.63 (Luminal B) and −1.02 (TNBC). LSM5 in the benign, luminal A and TNBC groups (log_2_ FC = −1.03, −0.89 and −1.31) was downregulated in this order. Finally, LSM3 in the TNBC group (log_2_ FC = −0.72) was downregulated (Figure 1 and Figure 2, Table S1).

### 2.3. Orbitrap-MS Analysis and Pathway Enrichment Analysis of the Differential Blood Proteins in CD8^+^ T Lymphocytes

**Figure 3:**
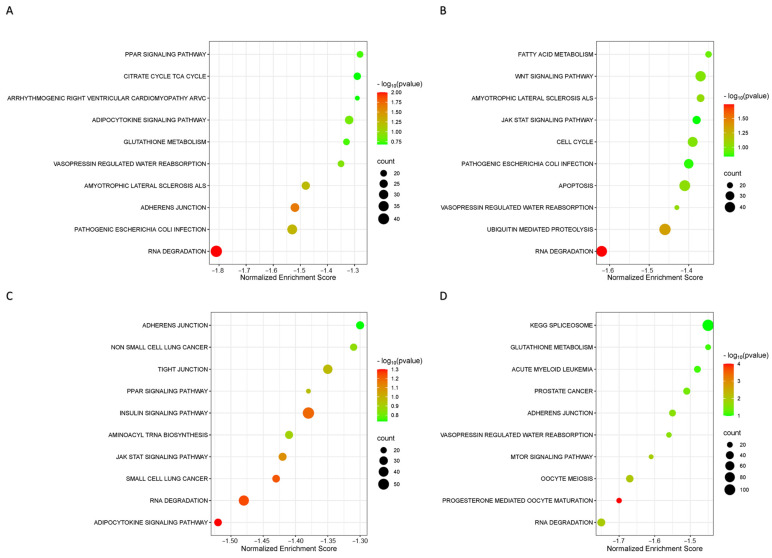
Bubble plots from the Kyoto Encyclopedia of Genes and Genomes (KEGG) of differentially expressed genes (DEGs) of the CD8 T lymphocytes obtained among the different groups, i.e., (**A**) benign, (**B**) luminal A, (**C**) luminal B and (**D**) TNBC vs. healthy controls. The color indicates the *p*-value (from the lowest in green to the highest in red), and the bubble size indicates the number of genes. The rich factor represents the proportion of the total number of genes.

**Figure 4:**
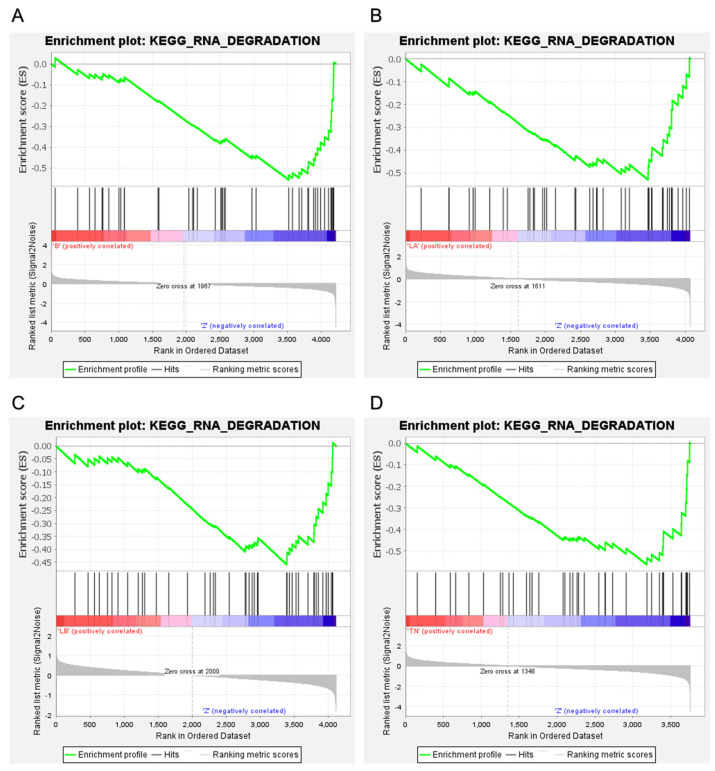
GSEA analysis of quantified proteins in CD8^+^ T lymphocytes in benign (**A**), luminal A (**B**), luminal B (**C**) and TNBC (**D**). Enrichment plot: KEGG RNA degradation; benign (NES = −1.80, *p* < 0.01), luminal A (NES = −1.62, *p* = 0.02), luminal B (NES = −1.47, *p* = 0.05) and TNBC (NES = −1.75, *p* = 0.01).

**Figure 5:**
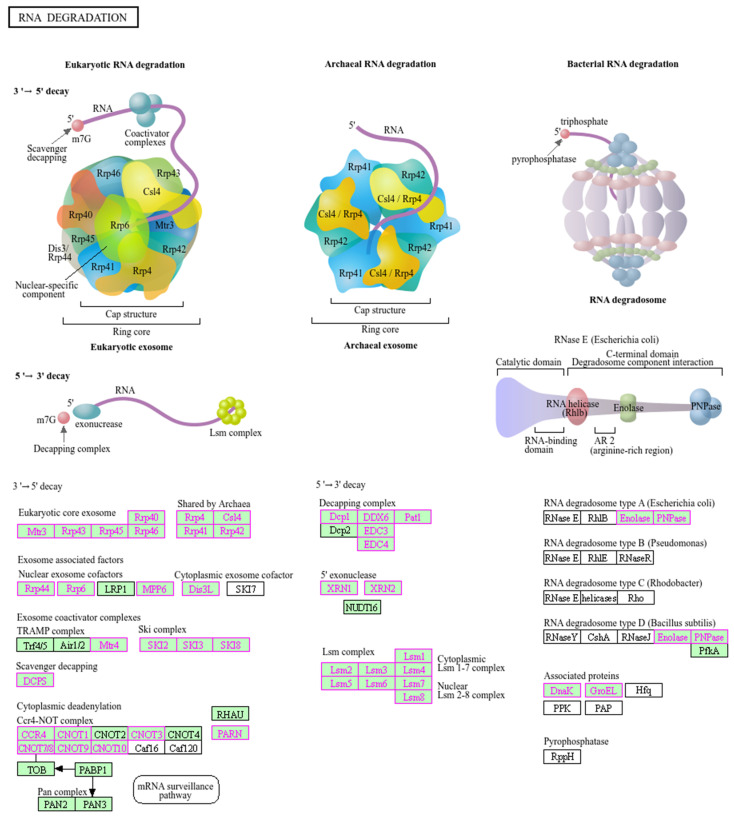
A total of 59 proteins involved in RNA degradation (KEGG pathway: hsa03018). Pink colored boxes represent 44 differentially expressed proteins found in this pathway (https://www.genome.jp/pathway/map03018, accessed on 13 March 2024).

The biological function and key pathway enrichment of the gene set between the benign, luminal A, luminal B and TNBC groups versus the healthy control in the peripheral blood CD8 T lymphocyte samples were analyzed using GSEA in the KEGG database. As a result, three significantly enriched pathways in the benign group (Figure 3A), two significantly enriched pathways in luminal A (Figure 3B), two significantly enriched pathways in luminal B (Figure 3C) and seven significantly enriched pathways in TNBC (Figure 3D) were obtained. The most significant in all groups was the RNA degradation pathway, i.e., in the benign group (NES = −1.80, *p* < 0.01), luminal A (NES = −1.62, *p* = 0.02), luminal B (NES = −1.47, *p* = 0.05) and TNBC (NES = −1.75, *p* = 0.01). We observed that the RNA degradation pathway in all groups was downregulated (Figure 4). Of the total 44 proteins from the CD8^+^ T lymphocytes in the RNA degradation pathway (Figure 5), 43 proteins in the benign group, 43 proteins in the luminal A group, 43 proteins in the luminal B group, and 40 proteins in the TNBC group using GSEA, were differentially expressed. There were 18 core enrichment proteins found in the benign group, i.e., a subset of proteins that contributed most to RNA degradation pathway enrichment (SKIC8, LSM8, LSM3, EXOSC5, CNOT3, DDX6, EXOSC9, LSM1, EXOSC4, EXOSC7, EXOSC3, LSM4, LSM2, EXOSC2, DIS3, EXOSC10, ENO2, LSM5 and EXOSC2). Similarly, we detected 16 core enrichment proteins in the luminal A group (CNOT10, DIS3, LSM4, EXOSC3, HSPD1, CNOT7, EDC4, LSM5, LSM2, LSM1, DDX6, DCP1A, ENO2, EXOSC2, EXOSC5 and EXOSC10). Likewise, in the luminal B group there were 17 core enrichment proteins (CNOT9, CNOT3, EDC4, DCP1A, LSM1, XRN1, EXOSC7, LSM5, CNOT6L, DIS3, DDX6, EXOSC9, EXOSC5, LSM2, EXOSC10, PATL1 and EXOSC2). Lastly, in the TNBC group, we identified 14 core enrichment proteins (EXOSC7, CNOT3, EXOSC2, LSM3, DIS3, EXOSC9, SKIC8, CNOT7, LSM2, DCP1A, LSM5, EXOSC10, EXOSC3 and ENO2) (Table S2).

## 3. Discussion

The main lymphocyte type in cell-mediated immunity and a key player in the immune responses linked to tumors is the CD8^+^ T lymphocyte [23]. Peripheral blood CD8^+^ T lymphocytes have various benefits over tumor-infiltrating lymphocyte (TILs) cells, including minimal invasiveness, high homogeneity, simple operation, easy access, and dynamic monitoring [24]. In comparison with different breast cancer subtypes, several studies reported that CD8^+^ T lymphocytes were more abundant [25] and more active [26] in TNBCs than in luminal tumors.

Cytotoxic CD8^+^ T lymphocytes are part of the adaptive immune system and play an important role in cancer-related processes. Cells presenting foreign antigens can be recognized by cytotoxic T lymphocytes via a specific interaction. This interaction causes the activated T lymphocytes to release proteins, including perforin 1 and granzymes which cause cytotoxic activity in tumor cells through membranolysis [27].

Perforin 1 is glycoprotein that is expressed only in cytotoxic T lymphocytes and forms a pore structure on the target-cell membrane to facilitate the entry of granzymes [28]. Natural killer (NK) cells and CD8^+^ T lymphocytes are the main source of perforin 1 [29]. In our study, a decrease in PRF1 in the benign group (log_2_ FC = −0.52) in comparison to the healthy control was observed.

**Figure 6:**
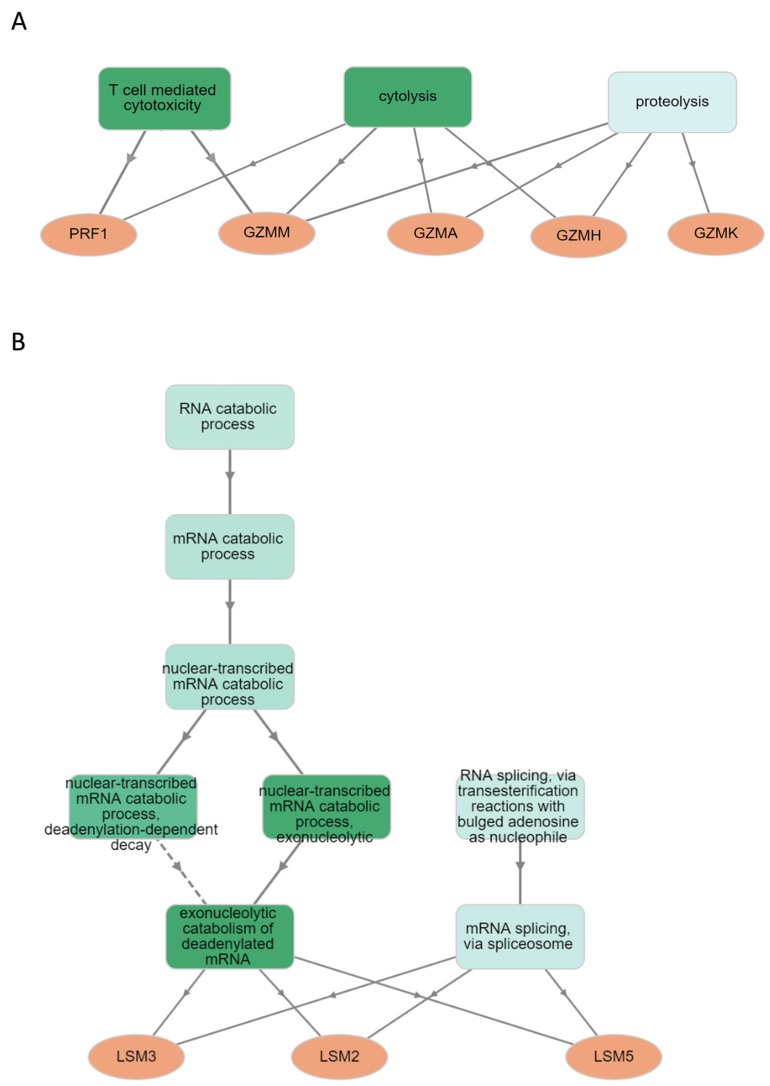
Gene ontology (GO) term enrichment analysis of genes (q-value threshold 0.05) using GOnet server (http://tools.dice-database.org/GOnet/, accessed on 14 May 2024). GO trees were built using biological process terms in a hierarchical layout. (**A**) T cell-mediated cytotoxicity, cytolysis and proteolysis (**B**) RNA catabolic process.

Granzymes are a highly conserved set of serine human proteases [30]. The most abundant and expressed granzymes in CD8^+^ T lymphocytes, irrespective of the activation state, are GZMA and GZMB [31]. There are conflicting findings about the process of cell death that GZMA causes, i.e., its function is not completely defined [32]. In our study, a decrease in GZMA in the luminal A group (log_2_ FC = −0.49) and the benign group in comparison to the healthy control was recorded. According to reports, human cytotoxic T lymphocytes constitutively and abundantly express GZMH, which has chymotrypsin-like (chymase) enzymatic activity. Human GZMH kills target tumor cells, leading to DNA and mitochondrial damage and the production of reactive oxygen species (ROS) [33]. Tahbaz and colleagues found that the GZMH level was significantly lower in patients than in the control group [34]. This result is consistent with the observation of Razavi and colleagues who reported lower GZMH levels in cancer patients before chemotherapy when compared to healthy controls [35]. Also, in our paper, a decrease in GZMH protein in the benign and luminal B groups (log_2_ FC = −0.65 and −0.46) in comparison to the healthy control, was noted in this order. GZMK and GZMA are similar in that they are closely linked to the same chromosome and they are the only granzymes that display tryptase-like activity, i.e., both granzymes cleave after basic residues, according to Arg and Lys [36]. Although some research has shown that GZMK can use perforin to initiate target cell death [37], other studies claim that GZMK kills target cells by targeting endoplasmic reticulum protein complexes, inducing mitochondrial damage, and starting caspase-independent cell death [38,39]. Recent studies have associated GZMK expression with memory CD8^+^ T lymphocytes [40]. In our study, an increase in GZMK (log_2_ FC = 0.72) in the TNBC group in comparison to the healthy control was detected. In target human cells, GZMM can cause cytotoxicity [41]. It has been demonstrated that GZMM may cause apoptotic cell death in two different ways: caspase-dependent and caspase-independent [42,43]. In our study, a decrease in GZMM (log_2_ FC= −0.56) in the benign group in comparison to the healthy control was observed. The proteins mentioned above (PRF1, GZMA, GZMH, GZMK and GZMM) that were significantly altered (log_2_ FC ≥ 0.38 or ≤−0.38, adjusted *p*-value < 0.05) participate in T cell-mediated cytotoxicity, cytolysis and proteolysis (Figure 6A).

Some mechanisms of mRNA decay control mRNA stability by dynamically varying protein levels. Mainly, the exonuclease and endonuclease pathways make up these “active” RNA degradation mechanisms [44,45]. In order to shorten the polyA tails of mRNAs, deadenylase complexes such PAN2-PAN3 and CCR4-NOT as well as PARN are drawn to mRNAs in the exonuclease pathway. Following the shortened polyA tails, the decapping enzymes DCP2 and NUDT16 are recruited by the LSM1–7/PAT1 complex through their interaction with 3′-terminal adenosine extensions. Exonuclease XRN1 from the 5′ ends of mRNAs and exosomes from the 3′ ends undergo degradation after decapping. Also, mRNAs are recruited by endonuclease, which produces unprotected 5′ and 3′ ends. XRN1 and exosomes recognize these unprotected ends and degrade them [46] (Figure 5).

Protein abundance is decreased by mRNA decay mechanisms more quickly than transcriptional suppression. However, once proteins are generated, indirect protein degradation through mRNA decay processes ought to occur more slowly than direct protein degradation. For example, proteins are approximately 5000 times more abundant in naïve T cells and 3000 times more abundant in 24 h-activated T cells than transcripts [47]. The ability of naïve T cells to maintain the expression of repressed mRNAs offers them the advantage of being able to start protein synthesis right away. Also, they contain many untranslated copies of mRNAs that are translated right away after their activation [20]. Thus, we suppose that the downregulation of the RNA degradation pathway might be a key step in the regulation of gene expression during phase transition, i.e., development, resting, activation and effector function T cells. In our study, in CD8^+^ T lymphocytes, the RNA degradation pathway was most significant in all groups, i.e., in the benign group (NES = −1.80, *p* < 0.01), luminal A (NES = −1.62, *p* = 0.02), luminal B (NES = −1.47, *p* = 0.05) and TNBC (NES = −1.75, *p* = 0.01). Also, the RNA degradation pathway in all groups was downregulated. We focused on LSM2, LSM3 and LSM5 proteins that were significantly altered (log_2_ FC ≥ 0.38 or ≤−0.38, adjusted *p*-value < 0.05), participating in the RNA degradation pathway (Figure 6B). RNA-binding Smith-like proteins (LSM1–LSM14B) are found in nearly all cellular organisms [48]. In our paper, LSM2 in the benign group (log_2_ FC = −0.59, luminal B group (−0.63) and TNBC group (−1.02 ) was downregulated. LSM5 in the benign, luminal A and TNBC groups (log_2_ FC = −1.03, −0.89 and −1.31) was downregulated in this order. Finally, LSM3 (log_2_ FC = −0.72) in the TNBC group was downregulated.

Although our findings are limited by the relatively small patient cohort and heterogeneity of CD8^+^ T lymphocytes, to our knowledge, it is the first study to employ label-free proteomic analysis of CD8^+^ T lymphocytes from peripheral blood in breast cancer subtype patients.

## 4. Materials and Methods

### 4.1. Patient’s Selection

Peripheral blood samples from newly diagnosed breast cancer (BC) patients taken prior to any therapy, to minimize therapy-induced proteomic alterations in BC subtypes, were used. Informed consent (2020/EK/06407) was obtained in written form from women both in the control group, as well as the breast cancer group (comprising the luminal A, luminal B, and TNBC groups). Randomized peripheral blood samples from the control group were obtained from the Department of Gynaecology and Obstetrics, and the samples from breast cancer groups were taken from the 1st Department of Surgery of the Faculty of Medicine, Pavol Jozef Šafárik University in Košice and the L. Pasteur University Hospital in Košice. All samples were taken during the period from June 2022 to March 2023. The 20 healthy control samples had a mean age of 68 years (in the range of 39–85 years), 8 samples from benign patients had a mean age of 53 years (between 39 and 73 years), 8 samples from luminal A patients had a mean age of 69 years (between 57 and 76 years), 8 samples from patients with luminal B breast cancer had a mean age of 61 years (ranging from 46 to 81 years) and 3 samples were taken from patients with TNBC with a mean age of 72 years (from 57 to 83 years). The clinico-pathological characteristics of patients with benign conditions and different breast cancer (BC) subtypes, luminal A, luminal B and triple-negative breast cancer (TNBC), are listed in Table 1. Inclusion criteria for control group were as follows: participants were healthy individuals with no history of breast cancer or any other malignancies (mammographic and ultrasonographic measures), biochemic, inflammatory and hematologic blood tests of all components were in physiological range, normal BMI (18.5–24.9). Exclusion criteria for the control group were as follows: pregnancy, smokers, chronic diseases (e.g., diabetes, cardiovascular diseases, autoimmune disorders), severe allergies or asthma, recent infections or vaccinations, COC (combined oral contraception), HRT (hormonal replacement therapy), presence of oncomarkers (CA 15-3, CA 27.29) and BRCA 1, BRCA 2 gene mutations.

### 4.2. Immunohistochemistry

Patient selection according to the immunohistochemistry (IHC) results was performed using an accredited medical laboratory, number of registration 158/Q-044, certified according to ISO 9001:2025, with a valid certificate until April 2026. Accreditation was carried out by SNAS (Slovak National Accreditation Service), which is a signatory of EA MLA and ILAC MRA in the field of the accreditation of medical laboratories. Molecular classification of BC by means of immunohistochemistry defines specific subtypes, such as luminal A (ER+, PR+, HER2−, Ki67 < 14%), luminal B (ER+, PR+, HER2−, Ki67 ≥ 14%) and triple negative (ER−, PR−, HER2−, high Ki67) [49]. The clinico-pathological data on patient and tumor characteristics were collected, including age, histological grade, pathological tumor size (pT), pathological node stage (pN), lymph node ratio, prognostic stage, metastasis, proliferation index (Ki-67), ER and PR expression (Table 1). Pathology tumor (pT), pathology node (pN) and prognostic stages were identified according to the eighth edition of the AJCC cancer staging manual [50].

### 4.3. Blood Sampling

Peripheral blood samples of 10 mL volume each were collected in BD Vacutainer K_2_EDTA tubes, cooled to 4 °C, and processed within 30 min of acquisition. A total of 10 mL of peripheral blood was diluted with 20 mL of saline solution (PBS containing BSA and EDTA, pH = 7.4) and subsequently centrifuged at 600× *g* for 10 min at 4 °C. The upper layer of plasma was removed and supplemented with an isolation solution so that the resulting volume was 10 mL.

### 4.4. Isolation of CD8^+^ T-Lymphocytes

A total of 250 μL of washed CD8^+^ magnetic beads (Dynabeads™ CD8^+^, Invitrogen by Thermo Fisher Scientific, Waltham, MA, USA) was added to the diluted blood. Subsequently, the samples were mixed in a multi-rotator (Multi Bio RS-24, Biosan, Riga, Latvia) at 4 °C for 20 min. A test tube with the sample and magnetic beads was inserted into a stand with a magnet (DynaMag™-50 Magnet, Invitrogen by Thermo Fisher Scientific, Waltham, MA, USA). The supernatant was carefully pipetted into a new 50 mL test tube. The captured magnetic beads were washed 3 times with the isolation solution. After the last removal of the isolation solution, CD8 T cells were lysed by adding 50 mM NH_4_HCO_3_ (200 μL). Subsequently, they were sonicated in an ultrasonic bath (15 min, frequency: 37 kHz, power: 90%, temperature: 4 °C), vortexed (5 min, 4 °C), sonicated in an ultrasonic block (amplitude: 85%, cycle: 0.5 s, temperature: 4 °C) for 15 min, and vortexed (5 min, 4 °C). Tubes were placed in a magnetic stand (2 min), and then the supernatant was collected.

### 4.5. In Solution Digestion

Protein concentration was determined using the BCA method QuantiPro BCA Assay Kit (Sigma-Aldrich, St. Louis, MO, USA). The required volumes of 0.025 M DTT solution (purity ≥ 98%, Biorad, Hercules, CA, USA) were added and the samples were then incubated in a thermomixer at 60 °C for 30 min. Subsequently, the required volumes of 0.25 M IAA solution (purity ≥ 99%, Biorad, Hercules, CA, USA) were added, after which the samples were incubated in a thermomixer in the dark, at 21 °C, for 30 min. The required volume of 5 mM CaCl_2_ (purity ≥ 98%, Merck, Darmstadt, Germany) solution was added and followed with trypsin (Roche) in a ratio of trypsin/sample = 1:40 (*w*/*w*). The samples were incubated at 21 °C overnight and then acidified to pH 3 by adding 20% formic acid (Merck, Darmstadt, Germany). The sample volume was adjusted using SpeedVac (Labconco, Kansas City, MO, USA). Samples were centrifuged at 18,000× *g*, at 4 °C.

### 4.6. Proteomics Analysis: Nano-UHPLC and ESI-MS

Processed samples were measured using a Thermo Scientific™ Orbitrap Exploris™ 480 high-resolution mass spectrometer coupled to a Thermo Scientific™ Vanquish™ Neo UHPLC System nanoliquid chromatograph. A Thermo Scientific™ EASY-Spray™ nano-spray injection (NSI) source was used as the ion source. A total of 10 µL of the sample with a concentration of 40 ng/µL was injected onto a trap column (PepMap™ Neo Trap Cartridge, Thermo Fisher Scientific, Waltham, MA, USA) with a length of 5 mm, a C_18_ particle size of 5 µm, and a flow rate of 60 µL/min. The peptides captured on this column were subsequently eluted using a preset time-dependent gradient onto an analytical column with a length of 50 cm and a C_18_ particle size of 2 µm, which was heated to 40 °C during the entire measurement using an integrated heater. During the gradient, two mobile phases were used, phase A: with a composition of 100% H_2_O, 0.1% formic acid and phase B: 80% acetonitrile, 20% H_2_O, 0.1% formic acid. After separation on the analytical column, the peptides at the emitter end of the EASY-Spray™ column were ionized with a voltage of 2000 V and sprayed into the mass spectrometer with the following set parameters: positive ion charge, for basic mass spectrometry (MS) scans on the orbitrap analyzer, a resolution of 120,000 at 200 *m*/*z* was used with a maximum injection time of 100 ms. Precursors for tandem mass spectrometry MS/MS scans with a set resolution of 30,000 *m*/*z* were selected based on the preset filters used, a charge in the range of 2–5, and an intensity cutoff of at least 1 × 10^4^. Fragmentation during MS/MS scans was ensured by collisions with inert gas molecules, in our case, with nitrogen molecules, using the Higher Energy Collisional Dissociation (HCD) technique in the collision cell of the spectrometer. The maximum time of injection into the orbitrap analyzer during the MS/MS scan was 50 ms.

### 4.7. Identification and Label-Free Protein Quantification

All raw files acquired from LC/MS runs were analyzed using the Proteome Discoverer (PD) version 2.5 (Thermo Scientific™) software. Protein identification was performed using a combination of three search engines available in PD, specifically, MS Amanda 2.0, SequestHT, and Mascot utilizing Uniprot Homo sapiens database (release 2023_02 which contained 182,026 sequences) for MS Amanda and SequestHT and Swiss-Prot Homo Sapiens database for Mascot. Proteolytic enzyme specification during the search was trypsin/P, cleaving after lysine (K) and arginine (R) except when followed by proline (P). Carbamidomethylation of cysteine was selected as a fixed modification, and oxidation of methionine, N-terminal acetylation, and Met-loss + acetylation of methionine were selected as variable modifications. Mass tolerances for precursor and fragment ions were set at 10 ppm and 0.02 Da, respectively. The minimum cutoff for peptide length was set at six amino acids, and the maximum permissible missed cleavage was set at two. The identified spectra were recorded using Percolator. Subsequently, filtering was applied at both the peptide spectrum match and peptide level, maintaining a false discovery rate (FDR) threshold of 1% for each match. At least 2 unique/razor peptides per protein identification were used for label-free quantification (LFQ) where the precursor abundance in the samples was compared based on the integration of intensities of the identified peptides. To determine the statistical significance of the LFQ results, a t-test was employed, enabling the identification of proteins that exhibited significant differences in abundance (0.77 ≥ abundance ratio ≥ 1.3; Benjamini–Hochberg adjusted *p*-value ≤ 0.05) within experimental conditions (Figure 1 and Figure 2 and Table S1).

### 4.8. Gene Set Enrichment Analysis

Gene set enrichment analysis (GSEA) was performed using GSEA v. 4.2.3 (Broad Institute, Cambridge, MA, USA, https://www.gsea-msigdb.org/gsea/index.jsp, accessed on 13 March 2024) on the gene set database https://data.broadinstitute.org/gsea-msigdb/msigdb/release/2023.2.Hs/ accessed on 13 March 2024) for the number of permutations (1000) and the permutation type (phenotype and gene set). RNA degradation pathways obtained were depicted using the Kyoto Encyclopedia of Genes and Genomes database (KEGG; https://www.kegg.jp/pathway/ko03018, accessed on 13 March 2024).

### 4.9. Bioinformatics Analysis

The label-free protein quantification analysis of CD8^+^ T lymphocytes in benign, luminal A, luminal B, and TNBC groups in comparison to the healthy control was performed. For the volcano plot analysis the following protein filter criteria were used: Master is equal to Master AND Unique Peptides is greater than or equal to 2 AND Abundances (Grouped) has any value in sample group AND Found in Sample Groups has confidence High (1% PSM FDR) in sample group AND Found in Samples has confidence High (1% PSM FDR) in at least 20 samples (Table S1). In addition, for the GSEA analysis, the following protein filter criteria were used: Master is equal to Master AND Unique Peptides is greater than or equal to 2 AND Abundances (Grouped) has any value in sample group AND Found in Sample Groups has confidence High (1% PSM FDR) in sample group (Table S3). To identify significantly enriched pathways, the FDR < 0.25 and nominal *p*-value < 0.05 criteria were determined using GSEA. All the MS proteomics data utilized in this study have been deposited to the ProteomeXchange Consortium via the PRIDE partner repository with the dataset identifier PXD051985.

## 5. Conclusions

Given the immunological role that T cells play in the treatment of disease, a better knowledge of RNA metabolism, i.e., the role that mRNA degradation pathways play in human physiology and pathology may lead to novel approaches to the therapeutic use of T cells.

## Figures and Tables

**Table 1:** The clinico-pathological characteristics of patients with benign conditions and different breast cancer (BC) subtypes: luminal A, luminal B and triple-negative breast cancer (TNBC).

Characteristic	Benign	Luminal A (*n*)	Luminal B (*n*)	TNBC (*n*)
Mean (IQR) age	53 (39–73)	69 (57–76)	61 (46–81)	72 (57–83)
Site, Left/Right	Left (8)	Left (3), Right (5)	Left (3), Right (5)	Left (2), Right(1)
Histologic type	Fibrosclerosis (4) Xantogranuloma(1) Ductus ectasia (1) Osseal metaplasis (1) Intracanalicular adenoma (1)	Invasive ductal carcinoma (8)	Invasive ductal carcinoma (8)	Invasive ductal carcinoma (3)
Histological grade	-	G1 (7), G2 (1)	G1 (3), G2 (5)	G3 (3)
Pathological tumour size	-	pT1 (5), pT2 (3)	pT1 (5), pT2 (3)	pT1 (1), pT2 (2)
Pathological node status	-	pN0 (7), pN1 (1)	pN0 (4), pN1 (1), pN2 (3)	pN0 (1), pN2 (2)
Lymph node ratio	-	≤20 (8)	≤20 (5), 0.21–0.65 (3)	≤20 (2), 0.21–0.65 (1)
Prognostic stage	-	IA (4), IIA (4)	IA (4), IIB (1), IIIA (3)	IA (1), IIIA (2)
Metastasis	-	Axillary lymph node metastasis (1)	Axillary lymph node metastasis (4)	Axillary lymph node metastasis (2)
Proliferation index (Ki-67)	-	<14% (8)	≥14% (8)	≥14% (3)
ER expression	-	90–100% (8)	30–95% (8)	-
PR expression	-	60–100% (8)	20–90% (8)	-

## Data Availability

The data presented in this study are available on request from the corresponding author.

## Supplementary Materials

The following supporting information can be downloaded at: https://www.mdpi.com/article/10.3390/ijms25126423/s1.
